# Iodophor-/H_2_O_2_-Mediated 2-Sulfonylation of Indoles and *N*-Methylpyrrole in Aqueous Phase

**DOI:** 10.3390/molecules29153564

**Published:** 2024-07-29

**Authors:** Yashuai Liu, Yutong Yuan, Jing He, Sheng Han, Yan Liu

**Affiliations:** 1 Basic Sciences Department, Shanxi Agricultural University, Jinzhong 030800, China; 2 The Key Laboratory for Green Processing of Chemical Engineering of Xinjiang Bingtuan, School of Chemistry and Chemical Engineering, Shihezi University, Shihezi 832004, China; hejing@shzu.edu.cn (J.H.); hansheng654321@sina.com (S.H.)

**Keywords:** iodophor, 2-sulfonylation, indoles, sulfonyl hydrazides

## Abstract

A convenient and efficient strategy for the preparation of 2-sulfonylindoles has been achieved through iodophor-/H_2_O_2_-mediated 2-sulfonylation of indoles with readily available sulfonyl hydrazides in the aqueous phase. Iodophor is commercially available and serves as the green catalyst and aqueous phase. A series of 2-sulfonylated products from indoles and *N*-methylpyrrole were synthesized in moderate yields in only 10 min. Control experiments were also conducted to reveal the mechanism of action. This method is environment friendly, easy to operate and suitable for a wide range of substrates.

## 1. Introduction

Indoles have emerged as a prominent structural motif in many natural products and pharmaceuticals [1,2,3,4,5,6,7]. Furthermore, the introduction of a sulfonyl moiety at the C2 position of the indole can often enhance its bioactivity [8,9]. In general, the C(2)–H sulfonylation of indoles has been the most straightforward way to synthesize 2-sulfonylindoles. However, in most of the literature, the sulfenylation of indoles often occurs at the C(3)–H position rather than the sulfonylation of C(2)–H [10,11,12,13,14]. And using the same strategy, 2-sulfenylindoles could be obtained when the C(3) position is occupied by substituents [15]. Thus, developing conditions for the direct synthesis of 2-sulfonylindoles is still a fascinating challenge. Over the past decade, numerous direct regioselective 2-sulfonylations of indoles with sodium sulfinate using molecular iodine and its salts as catalysts have been explored [16,17,18,19,20]. These reactions often require oxidants (e.g., TBHP and oxone) or promoters (e.g., TMSOTf). In 2017, Yu and co-workers developed an electrochemical 2-sulfonylation of 1*H*-indoles under chemical oxidant-free conditions, yielding various 2-sulfonylindoles in good to high yields [21]. In addition, *p*-toluenesulfonyl cyanide [22] and sulfonyl hydrazides [23,24] have also been used to construct 2-sulfonyl indoles. Sulfonyl hydrazides, in particular, have proved to be environmentally friendly sulfur sources for the sulfonylation of indoles through the cleavage of their C–N bonds [25,26,27,28,29,30]. However, in previous similar work [23,24], the solvents are usually organic, or stoichiometric iodine or iodide salts in the aqueous phase is required. The main reason for this is that iodine or iodine produced in situ is less soluble in water. The combination of povidone and iodine could increase the solubility of iodine and improve its catalytic efficiency. Iodophor (povidone-iodine in water) is inexpensive, commercially available and not harmful to the environment. As a disinfectant, iodophor is widely used in medical treatment and in our daily life. However, it has rarely been employed to catalyze organic reactions. Therefore, choosing iodophor as a green catalyst and aqueous phase for 2-sulfonylation of indoles with sulfonyl hydrazides is highly desirable. In this context, we report a fast, mild and efficient iodophor-catalyzed 2-sulfonylation of substituted indoles using 30% H_2_O_2_ solution as an oxidant in the aqueous phase. Furthermore, the synthetic strategy has a wide substrate scope with high tolerance to various functional groups and steric hindrance in indoles and sulfonyl hydrazides.

## 2. Results and Discussion

The reaction of 1*H*-indole (**1a**, 0.5 mmol) and *p*-toluenesulfonyl hydrazide (**2a**, 1.0 mmol) was chosen as a model reaction for optimization, and the results are summarized in Table 1. Initially, the reaction was conducted with 0.06 mL (1 equiv.) H_2_O_2_ and 2 mL iodophor (0.04 mmol I_2_) at 25 °C for 2 h, giving the desired product **3a** in only 28% yield (Table 1, entry 1). Moreover, 2-sulfonylation of indole could proceed rapidly, affording a similar yield of **3a** in 30% in only 10 min (Table 1, entry 2). Fortunately, increasing the amount of H_2_O_2_ solution (1 mL) further improved the reaction yield to 42% (Table 1, entry 3). Subsequently, a temperature range from 50 °C to 100 °C was investigated (Table 1, entries 4–8). The variations in temperature showed that 60 °C was optimal, giving 70% yield of **3a**. Reducing the I_2_ loading to 0.02 mmol resulted in a significantly lower yield (35%) (Table 1, entry 9). In addition, an alternative 70% TBHP solution was employed as an oxidant, showing less efficiency (Table 1, entry 10). For cost and environmental reasons, 30% H_2_O_2_ solution was reduced by half for the oxidative 2-sulfonylation. A relatively low yield was obtained (Table 1, entry 11). Meanwhile, we attempted to optimize the reaction at 25 °C or 90 °C, but this only resulted in lower yields of 38% and 32%, respectively. When the reaction of **1a** and **2a** in a 1:1 ratio was studied, it gave a comparatively lower yield of 51%. This result indicates that the sulfonyl hydrazides could not be completely converted to the sulfonyl radicals. Therefore, excess sulfonyl hydrazides are required in this reaction. 

Finally, the optimized reaction conditions are as follows: indole (**1a**) (0.5 mmol) with *p*-toluenesulfonyl hydrazide (**2a**) (1 mmol), H_2_O_2_ (1 mL) and iodophor (2 mL, 0.04 mmol I_2_) at 60 °C for 10 min.

**Figure 1:**
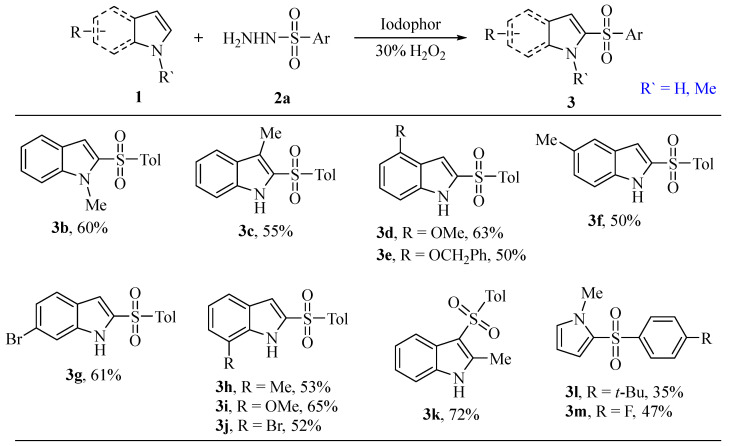
Scope of indoles ^1,2^. ^1^ Reaction conditions: **1** (0.5 mmol), **2a** (1 mmol), iodophor (2 mL), H_2_O_2_ (1 mL), under air, 60 °C, 10 min. ^2^ Isolated yield.

On the basis of optimal reaction conditions, the scope of sulfonyl indoles **1** and sulfonyl hydrazides **2** was investigated, respectively. First, a series of aryl-substituted indoles with electron-donating substituents (Me, OMe and OCH_2_Ph) were treated with *p*-toluenesulfonyl hydrazide (**2a**) to afford the corresponding products (**3b**~**3f**, **3h** and **3i**) in moderate yields (50~65%). The results are summarized in Figure 1. Among them, the substitution of OMe gave a slightly better reactivity than the other groups. In comparison, 6-bromo- and 7-bromo-indoles were employed to give the target products **3g** and **3j** in 61% and 52%, respectively. These results showed that the electronic effect of the substituents on the indole moiety has little significant impact on this synthetic method. Utilizing the same strategy, the 3-sulfonylation proceeded smoothly when the C-2 position was occupied by methyl, yielding the product **3k** in 72% yield. In addition, the 2-sulfonylation of *N*-methylpyrrole was also investigated. Generally, the 2-sulfonylation of *N*-methylpyrrole is conducted with reactive sulfur sources under harsh reaction conditions [20,31]. Fortunately, the 2-sulfonylation of *N*-methylpyrrole with 4-arylsulfonyl hydrazides could proceed smoothly, giving the corresponding products (**3l** and **3m**) in moderate yields. These results indicate that the synthetic strategy has a high tolerance to both electron-withdrawing groups and electron-donating groups of arylsulfonyl hydrazides **2**.

**Figure 2:**
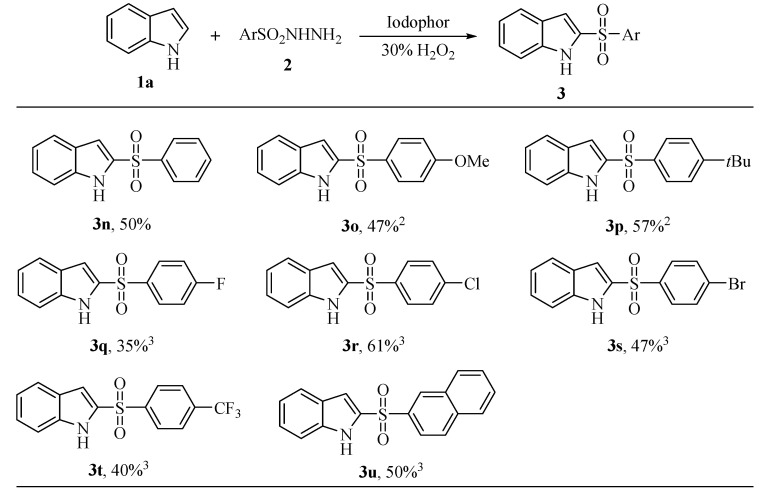
Scope of sulfonyl hydrazides ^1^. ^1^ Reaction conditions: **5a** (0.5 mmol), **2** (1 mmol), iodophor (2 mL), H_2_O_2_ (1 mL), under air, 60 °C, 10 min. ^2^ Reaction time 2 h, 25 °C. ^3^ Reaction time 5 h, 25 °C.

Subsequently, the scope of sulfonyl hydrazides was also evaluated (Figure 2). It was disappointing that various substrates with functional groups such as methoxy, *t*-Bu, halogen and CF_3_ in the aromatic rings were not applicable to the optimal reaction conditions. Only when benzenesulfonohydrazide was employed could the target product **3n** be obtained in 50% yield. The temperature was found to be crucial for the 2-sulfonylation of arylsulfonyl hydrazides. When the processes were carried out at 25 °C, the corresponding products (**3o**~**3t**) were obtained in moderate yields. And as shown in Figure 2, arylsulfonyl hydrazides bearing electron-withdrawing groups showed better reactivity and gave the desired products in only 2 h, whereas the reaction of arylsulfonyl hydrazides with electron-donating groups should proceed for 5 h to give moderate yields. Naphthalene-2-sulfonohydrazide was also employed to afford the product **3u** in 50% yield.

**Scheme 1:**
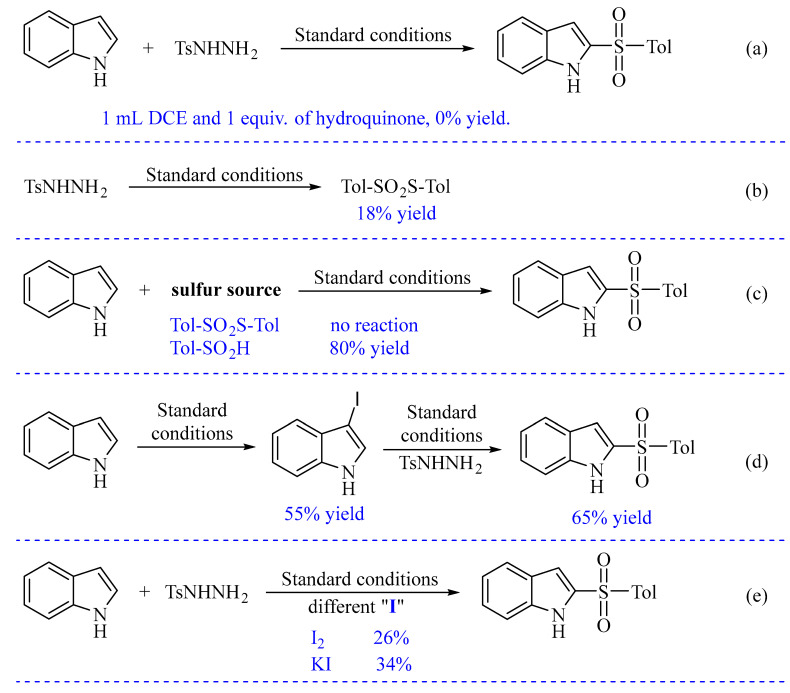
Control experiments. (**a**) Reactions in the presence of radical trapping reagents. (**b**) Reactivity of TsNHNH_2_ alone. (**c**) Reaction of iodole with other sulfur. (**d**) The stepwise reaction of iodole with iodophor and TsNHNH_2_ in succession. (**e**) The reaction of iodole and TsNHNH_2_ catalyzed by I_2_ and KI.

To further understand the mechanism of this transformation, a series of control experiments were carried out. First, 1 equiv. of hydroquinone was used as a radical scavenger in the 2-sulfonylation of 1*H*-indole (**1a**) with *p*-toluenesulfonyl hydrazide (**2a**), and dichloroethane was also added to increase the solubility of hydroquinone. It was found that the reaction did not proceed (Scheme 1a), suggesting that the reaction is likely to be a radical process. Self-coupling of *p*-toluenesulfonyl hydrazide occurred in the absence of 1*H*-indole, giving the corresponding product S-*p*-tolyl 4-methylbenzenesulfonothioate in only 18% (Scheme 1b). Subsequently, S-*p*-tolyl 4-methylbenzenesulfonothioate was treated with 1*H*-indole, and no product was detected (Scheme 1c). The results indicate that S-*p*-tolyl 4-methylbenzenesulfonothioate is not involved as an intermediate in 2-sulfonylation. When sodium 4-methylbenzenesulfinate was used as a sulfur source, the reaction proceeded to the 2-sulfonylated product in 80% yield (Scheme 1c). In the absence of *p*-toluenesulfonyl hydrazide, 1*H*-indole was iodinated by stoichiometric iodophor to give 3-iodo-1*H*-indole in a 55% yield (Scheme 1d). In addition, 3-iodo-1*H*-indole could be further reacted with *p*-toluenesulfonyl hydrazide to give the 2-sulfonylated product (**3a**) in a 65% yield (Scheme 1d). Finally, the control experiments with the same catalytic loading of iodine or NaI in H_2_O were investigated and gave lower yields of 26% and 34%, respectively (Scheme 1e). During the reaction, we found that iodine or iodine produced in situ was less soluble in the reaction solution. The result suggests that the combination of povidone and iodine could increase the solubility of iodine and improve its catalytic efficiency. All of the above reactions were conducted under the standard conditions: indole (0.5 mmol) with *p*-toluenesulfonyl hydrazide or another sulfur source (1 mmol), H_2_O_2_ (1 mL) and iodophor (2 mL, 0.04 mmol I_2_) at 60 °C for 10 min.

**Scheme 2:**
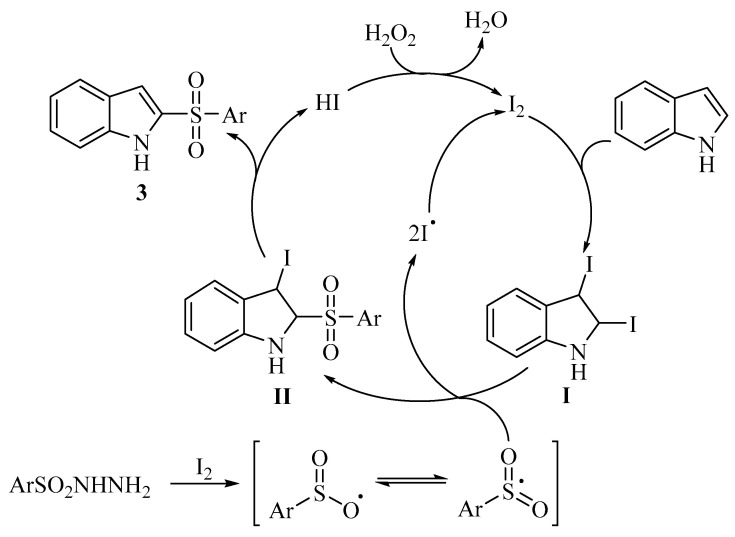
Proposed mechanism for iodophor-mediated 2-sulfonylation of indoles.

Based on the results of control experiments and the existing literature [18,19,23], a plausible mechanism for iodophor-mediated 2-sulfonylation of indoles is illustrated in Scheme 2. Molecular iodine derived from iodophor is added to indole to form the important intermediate 2,3-diiodoindoline (**I**). Meanwhile, molecular iodine also rapidly activates *p*-toluenesulfonyl hydrazide to give the sulfonyl radical. Afterwards, the reaction of intermediate **I** with the sulfonyl radical leads to the formation of intermediate **II** and an iodine radical. Intermediate **II** undergoes an HI elimination to give the 2-sulfonylated product (**3**). And the molecule iodine in the catalytic system can be regenerated from the oxidation reaction of HI by H_2_O_2_ or coupling of two iodine radicals.

## 3. Materials and Methods

### 3.1. General Methods 

Unless otherwise stated, all reactions were carried out in Schlenk tubes. Melting points were determined using a melting point apparatus and are uncorrected. Chemicals were purchased commercially and were used without further purification. Column chromatography was performed on Qingdao Ocean Chemical silica gel (Qingdao, China) (200~300 mesh). ^1^H NMR and ^13^C NMR spectra were recorded on a Bruker Avance III HD 400 MHz spectrometer (Bruker, Ettlingen, Germany) in CDCl_3_ with tetramethylsilane (TMS) as the internal standard. 

### 3.2. General Procedure for Iodophor-/H_2_O_2_-Mediated 2-Sulfonylation of Indoles and N-Methylpyrrole

Indole **1a** (0.5 mmol) and benzenesulfonyl hydrazide **2a** (1.0 mmol) were placed in a sealed 10 mL reaction tube, and 2 mL iodophor (5% solution of the povidone-iodine in water) (0.04 mmol I_2_) and 1 mL 30% H_2_O_2_ solution were added. Then, the reaction proceeded at 60 °C for 10 min. After the reaction finished, saturated salt solution (10 mL) was used and extracted with ethyl acetate (3 × 10 mL). The combined organic layers were dried over anhydrous Na_2_SO_4_, and the organic solvent was evaporated on a rotatory evaporator. The crude product was purified by flash chromatography on silica gel (PE/EtOAc) to give the corresponding product **3a**.

### 3.3. The Characterization Data of Products

*2-tosyl-1H-indole* (3a) [23] (see Supplementary Materials)

^1^H NMR (400 MHz, CDCl_3_) *δ* 9.02 (s, 1H), 7.99 (d, *J* = 8.4 Hz, 2H), 7.77 (d, *J* = 8.1 Hz, 1H), 7.52 (d, *J* = 9.2 Hz, 1H), 7.46–7.40 (m, 3H), 7.31–7.25 (m, 2H), 2.50 (s, 3H).

*1-methyl-2-tosyl-1H-indole* (**3b**) [23]

^1^H NMR (400 MHz, CDCl_3_) *δ* 7.77 (d, *J* = 8.3 Hz, 2H), 7.62 (d, *J* = 8.1 Hz, 1H), 7.32–7.21 (m, 5H), 7.10 (t, *J* = 7.4 Hz, 1H), 3.77 (s, 3H), 2.33 (s, 3H).

*3-methyl-2-tosyl-1H-indole* (**3c**) [23]

^1^H NMR (400 MHz, CDCl_3_) *δ* 9.36-9.08 (m, 1H), 7.86 (d, *J* = 5.7 Hz, 2H), 7.58 (d, *J* = 8.1 Hz, 1H), 7.38 (d, *J* = 8.4 Hz, 1H), 7.33-7.27 (m, 1H), 7.25 (d, *J* = 8.1 Hz, 2H), 7.13 (t, *J* = 7.5 Hz, 1H), 2.52 (s, 3H), 2.36 (s, 3H).

*4-methoxy-2-tosyl-1H-indole* (**3d**) [23]

^1^H NMR (400 MHz, CDCl_3_) *δ* 8.92 (s, 1H), 7.89 (d, *J* = 8.4 Hz, 2H), 7.32–7.24 (m, 4H), 7.01 (d, *J* = 8.4 Hz, 1H), 6.54 (d, *J* = 7.8 Hz, 1H), 3.95 (s, 3H), 2.41 (s, 3H).

*4-(benzyloxy)-2-tosyl-1H-indole* (**3e**) [23]

^1^H NMR (400 MHz, CDCl_3_) *δ* 8.96 (s, 1H), 7.86 (d, *J* = 8.3 Hz, 2H), 7.46 (d, *J* = 7.2 Hz, 2H), 7.39 (t, *J* = 7.3 Hz, 2H), 7.33 (t, *J* = 7.2 Hz, 2H), 7.26 (d, *J* = 8.1 Hz, 2H), 7.21 (t, *J* = 8.1 Hz, 1H), 6.99 (d, *J* = 8.4 Hz, 1H), 6.57 (d, *J* = 7.8 Hz, 1H), 5.17 (s, 2H), 2.37 (s, 3H).

*5-methyl-2-tosyl-1H-indole* (**3f**) [23]

^1^H NMR (400 MHz, CDCl_3_) *δ* 9.01 (s, 1H), 7.87 (d, *J* = 8.3 Hz, 2H), 7.42 (s, 1H), 7.29 (d, *J* = 8.3 Hz, 2H), 7.26 (d, *J* = 2.7 Hz, 1H), 7.15 (d, *J* = 8.4 Hz, 1H), 7.09 (s, 1H), 2.41 (s, 3H), 2.38 (s, 3H).

*6-bromo-2-tosyl-1H-indole* (**3g**) [23]

^1^H NMR (400 MHz, CDCl_3_) *δ* 9.25 (s, 1H), 7.87 (d, *J* = 8.3 Hz, 2H), 7.54 (s, 1H), 7.49 (d, *J* = 8.6 Hz, 1H), 7.28 (d, *J* = 8.2 Hz, 2H), 7.25 (d, *J* = 8.6 Hz, 2H), 7.11 (s, 1H), 2.38 (s, 3H).

*7-methyl-2-tosyl-1H-indole* (**3h**) [23]

^1^H NMR (400 MHz, CDCl_3_) δ 9.02 (s, 1H), 7.92 (d, *J* = 8.3 Hz, 2H), 7.50 (d, *J* = 7.7 Hz, 1H), 7.30 (d, *J* = 8.4 Hz, 2H), 7.18 (s, 1H), 7.13–7.08 (m, 2H), 2.48 (s, 3H), 2.39 (s, 3H).

*7-methoxy-2-tosyl-1H-indole* (**3i**) [23]

^1^H NMR (400 MHz, CDCl_3_) *δ* 9.04 (s, 1H), 7.86 (d, *J* = 8.2 Hz, 2H), 7.26 (m, 3H), 7.13 (s, 1H), 7.08 (t, *J* = 7.9 Hz, 1H), 6.73 (d, *J* = 7.7 Hz, 1H), 3.94 (s, 3H), 2.38 (s, 3H).

*7-bromo-2-tosyl-1H-indole* (**3j**) [23]

^1^H NMR (400 MHz, CDCl_3_) *δ* 9.40 (s, 1H), 8.05 (d, *J* = 7.0 Hz, 2H), 7.58 (d, *J* = 7.6 Hz, 1H), 7.46 (d, *J* = 8.0 Hz, 1H), 7.42–7.27 (m, 3H), 7.15 (t, *J* = 7.9 Hz, 1H), 2.45 (s, 3H).

*2-methyl-3-tosyl-1H-indole* (**3k**) [23]

^1^H NMR (400 MHz, CDCl_3_) δ 9.18 (s, 1H), 7.89 (d, *J* = 7.2 Hz, 1H), 7.75 (d, *J* = 8.3 Hz, 2H), 7.17 (d, *J* = 7.0 Hz, 1H), 7.12 (d, *J* = 8.0 Hz, 2H), 7.18–7.03 (m, 2H), 2.56 (s, 3H), 2.25 (s, 3H).

*2-((4-(tert-butyl)phenyl)sulfonyl)-1-methyl-1H-pyrrole* (**3l**) [23]

^1^H NMR (400 MHz, CDCl_3_) *δ* 7.83 (d, *J* = 8.7 Hz, 2H), 7.54 (d, *J* = 8.7 Hz, 2H), 7.05 (dd, *J* = 4.0, 1.9 Hz, 1H), 6.78 (t, *J* = 2.2 Hz, 1H), 6.22–6.17 (m, 1H), 3.75 (s, 3H), 1.35 (s, 9H).

*2-((4-fluorophenyl)sulfonyl)-1-methyl-1H-pyrrole* (**3m**) [23]

^1^H NMR (400 MHz, CDCl_3_) δ 7.84–7.79 (m, 2H), 7.09 (t, *J* = 8.6 Hz, 2H), 6.93 (dd, *J* = 4.0, 1.9 Hz, 1H), 6.70 (t, *J* = 2.1 Hz, 1H), 6.09 (dd, *J* = 4.0, 2.6 Hz, 1H), 3.63 (s, 3H).

*2-(phenylsulfonyl)-1H-indole* (**3n**) [23]

^1^H NMR (400 MHz, CDCl_3_) δ 9.45 (s, 1H), 7.94 (d, *J* = 7.7 Hz, 2H), 7.57 (d, *J* = 8.0 Hz, 1H), 7.45 (t, *J* = 7.3 Hz, 1H), 7.40–7.33 (m, 3H), 7.22 (t, *J* = 7.7 Hz, 1H), 7.14 (s, 1H), 7.07 (t, *J* = 7.5 Hz, 1H).

*2-((4-methoxyphenyl)sulfonyl)-1H-indole* (**3o**) [23]

^1^H NMR (400 MHz, CDCl_3_) *δ* 9.12 (s, 1H), 7.94 (d, *J* = 8.8 Hz, 2H), 7.65 (d, *J* = 8.1 Hz, 1H), 7.41 (d, *J* = 8.3 Hz, 1H), 7.32 (t, *J* = 7.6 Hz, 1H), 7.20–7.12 (m, 2H), 6.95 (d, *J* = 8.8 Hz, 2H), 3.83 (s, 3H).

*2-((4-(tert-butyl)phenyl)sulfonyl)-1H-indole* (**3p**) [23]

^1^H NMR (400 MHz, CDCl_3_) *δ* 9.51 (s, 1H), 7.96 (d, *J* = 8.7 Hz, 2H), 7.66 (d, *J* = 8.1 Hz, 1H), 7.48 (d, *J* = 8.7 Hz, 2H), 7.43 (d, *J* = 7.7 Hz, 1H), 7.31 (t, *J* = 7.2 Hz, 1H), 7.22 (s, 1H), 7.16 (t, *J* = 7.1 Hz, 1H), 1.28 (s, 9H).

*2-((4-fluorophenyl)sulfonyl)-1H-indole* (**3q**) [23]

^1^H NMR (400 MHz, CDCl_3_) *δ* 9.09 (s, 1H), 7.99 (dd, *J* = 10.4, 6.4 Hz, 2H), 7.64 (d, *J* = 8.0 Hz, 1H), 7.39 (d, *J* = 8.3 Hz, 1H), 7.31 (t, *J* = 7.2 Hz, 1H), 7.17–7.11 (m, 4H).

*2-((4-chlorophenyl)sulfonyl)-1H-indole* (**3r**) [23]

^1^H NMR (400 MHz, CDCl_3_) *δ* 9.50 (s, 1H), 8.04 (d, *J* = 8.7 Hz, 2H), 7.49 (d, *J* = 8.8 Hz, 3H), 7.41 (d, *J* = 6.0 Hz, 2H), 7.27–7.22 (m, 2H).

*2-((4-bromophenyl)sulfonyl)-1H-indole* (**3s**) [23]

^1^H NMR (400 MHz, CDCl_3_) *δ* 8.90 (s, 1H), 7.78 (d, *J* = 8.7 Hz, 2H), 7.60 (d, *J* = 8.1 Hz, 1H), 7.56 (d, *J* = 8.7 Hz, 2H), 7.35 (d, *J* = 8.4 Hz, 1H), 7.32–7.25 (m, 1H), 7.12 (dd, *J* = 9.3, 4.6 Hz, 2H).

*2-((4-(trifluoromethyl)phenyl)sulfonyl)-1H-indole* (**3t**) [23]

^1^H NMR (400 MHz, CDCl_3_) *δ* 8.95 (s, 1H), 8.11 (d, *J* = 8.2 Hz, 2H), 7.75 (d, *J* = 8.3 Hz, 2H), 7.67 (d, *J* = 8.1 Hz, 1H), 7.42 (d, *J* = 8.4 Hz, 1H), 7.38–7.33 (m, 1H), 7.24 (d, *J* = 0.8 Hz, 1H), 7.19 (t, *J* = 7.5 Hz, 1H).

*2-(naphthalen-2-ylsulfonyl)-1H-indole* (**3u**) [23]

^1^H NMR (400 MHz, CDCl_3_) *δ* 8.89 (s, 1H), 8.59 (s, 1H), 7.95 (d, *J* = 7.5 Hz, 1H), 7.91 (s, 2H), 7.85 (d, *J* = 7.7 Hz, 1H), 7.61 (dt, *J* = 8.2, 7.1 Hz, 3H), 7.39 (d, *J* = 8.4 Hz, 1H), 7.31 (t, *J* = 7.7 Hz, 1H), 7.23 (s, 1H), 7.15 (t, *J* = 7.5 Hz, 1H).

## 4. Conclusions

In summary, we have developed an eco-friendly, fast and effective iodophor-/H_2_O_2_-mediated 2-sulfonylation of indoles with readily available sulfonyl hydrazides in the aqueous phase. Iodophor is commercially available and serves as the green catalyst and aqueous phase. In this approach, the 2-sulfonylation of indoles with sulfonyl hydrazides proceeded smoothly, yielding a series of 2-sulfonylated products in moderate yields in only 10 min. In addition, a series of control experiments were carried out to disclose the radical reaction mechanism of 2-sulfonylation.

## Figures and Tables

**Table 1:** Optimization of reaction conditions ^1^.

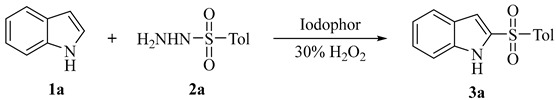					
**Entry**	**Oxidation**	**Iodophor** (**mL**)	**T** (**°C**)	**t** (**min**)	**Yield** (**%**) **^2^**
1	H_2_O_2_ (0.06 mL) ^3^	2 (0.04 mmol I_2_)	25	120	28
2	H_2_O_2_ (0.06 mL) ^3^	2 (0.04 mmol I_2_)	25	10	30
3	H_2_O_2_ (1 mL)	2 (0.04 mmol I_2_)	25	10	42
4	H_2_O_2_ (1 mL)	2 (0.04 mmol I_2_)	50	10	45
5	H_2_O_2_ (1 mL)	2 (0.04 mmol I_2_)	60	10	70
6	H_2_O_2_ (1 mL)	2 (0.04 mmol I_2_)	80	10	50
7	H_2_O_2_ (1 mL)	2 (0.04 mmol I_2_)	90	10	40
8	H_2_O_2_ (1 mL)	2 (0.04 mmol I_2_)	100	10	36
9	H_2_O_2_ (1 mL)	1 (0.02 mmol I_2_)	25	10	35
10	TBHP (1 mL)	2 (0.04 mmol I_2_)	60	10	55
11	H_2_O_2_ (0.5 mL)	2 (0.04 mmol I_2_)	60	10	58
12	H_2_O_2_ (0.5 mL)	2 (0.04 mmol I_2_)	25	10	38
13	H_2_O_2_ (0.5 mL)	2 (0.04 mmol I_2_)	90	10	32

^1^ Reaction conditions: **1a** (0.5 mmol), **2** (1 mmol), under air. ^2^ Isolated yields. ^3^ 1 equiv. H_2_O_2._

## Data Availability

Data are contained within the article.

## Supplementary Materials

The following supporting information can be downloaded at: https://www.mdpi.com/article/10.3390/molecules29153564/s1. The charts of ^1^H NMR of products.
