# Supplementary material for: Iodophor-/H2O2-Mediated 2-Sulfonylation of Indoles and N-Methylpyrrole in Aqueous Phase
Source: Molecules. 2024 Jul 29;29(15):3564. doi: 10.3390/molecules29153564 (PMC11314382; doi:10.3390/molecules29153564)

Iodophor/H_2O_2 mediated 2-Sulfonylation of Indoles and N-Methylpyrrole in Aqueous Phase

Supporting Information

Table of Contents

NMR spectra 2

NMR spectra

2-tosyl-1*H*-indole (3a)

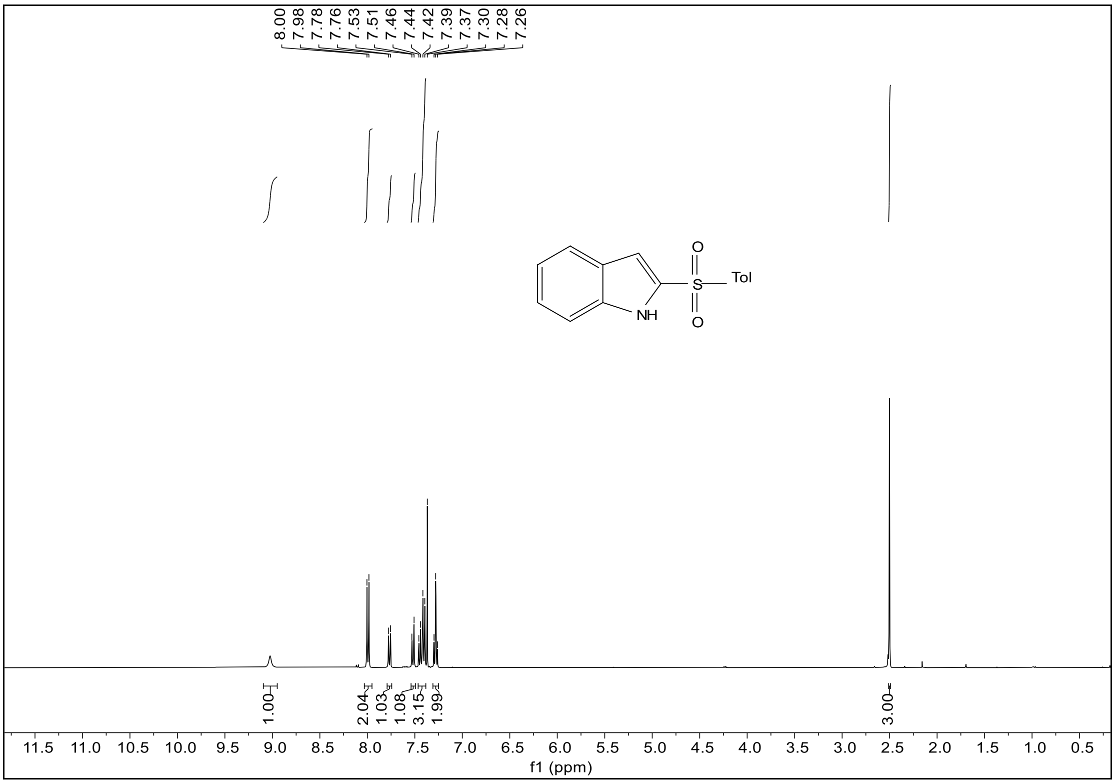

1-methyl-2-tosyl-1*H*-indole (3b)

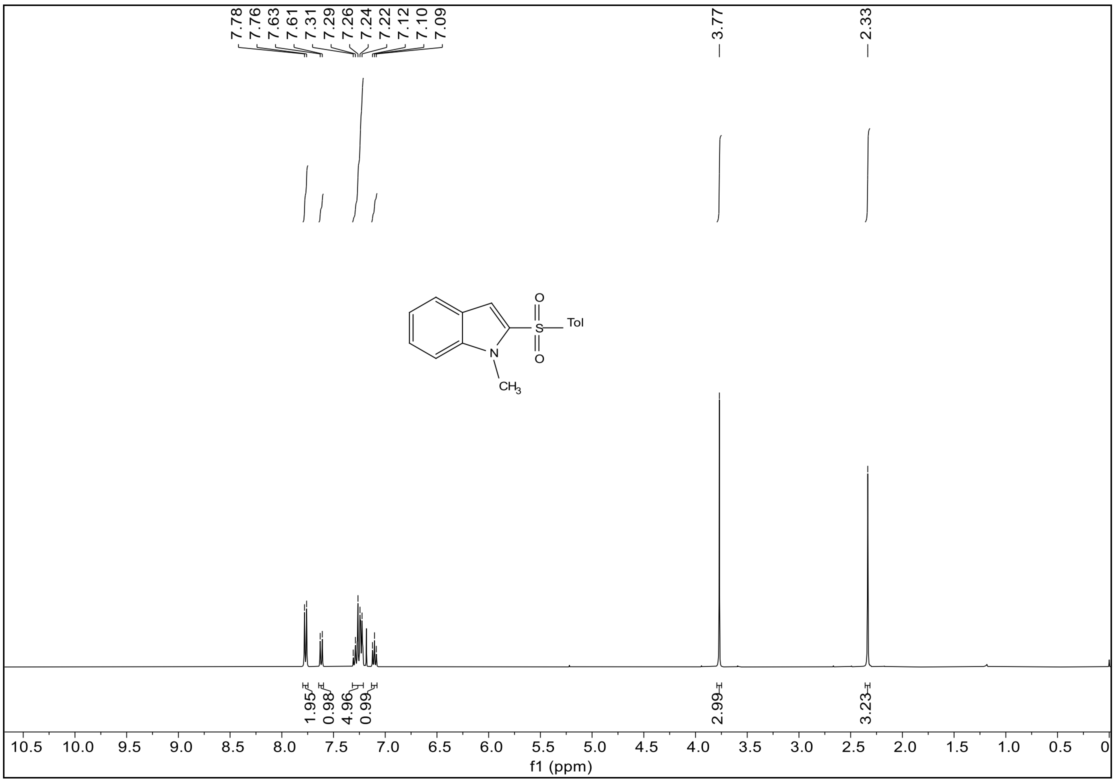

3-methyl-2-tosyl-1*H*-indole (3c)

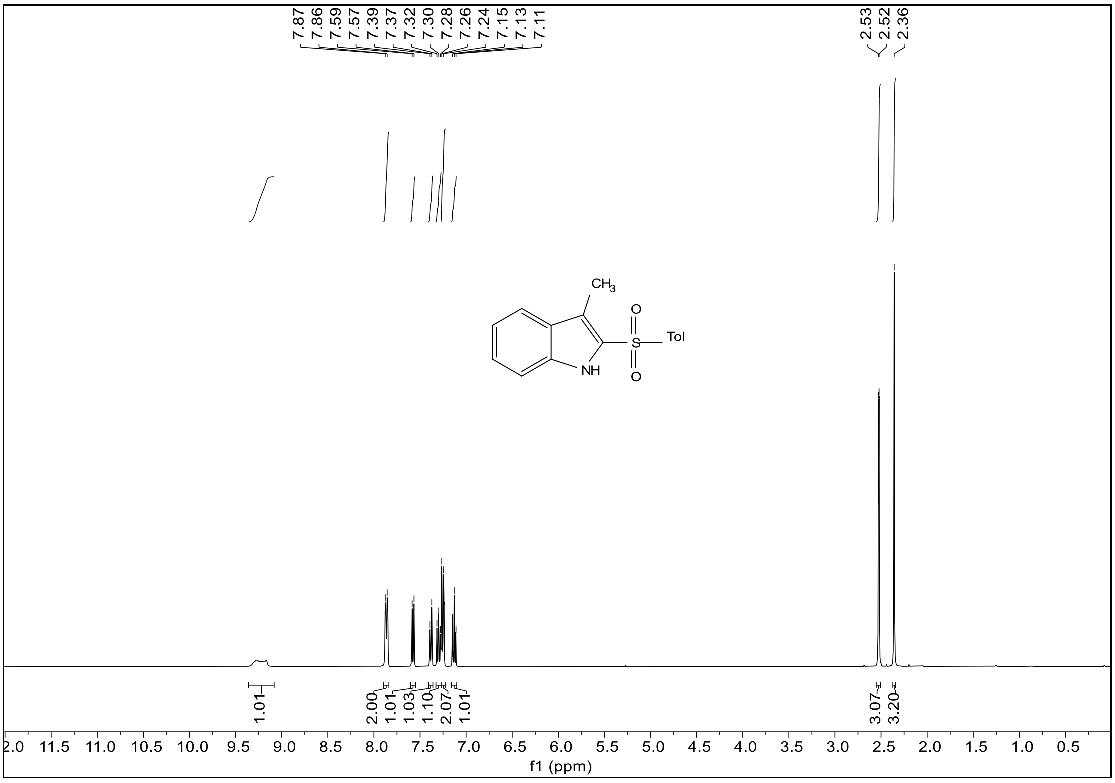

4-methoxy-2-tosyl-1*H*-indole(3d)

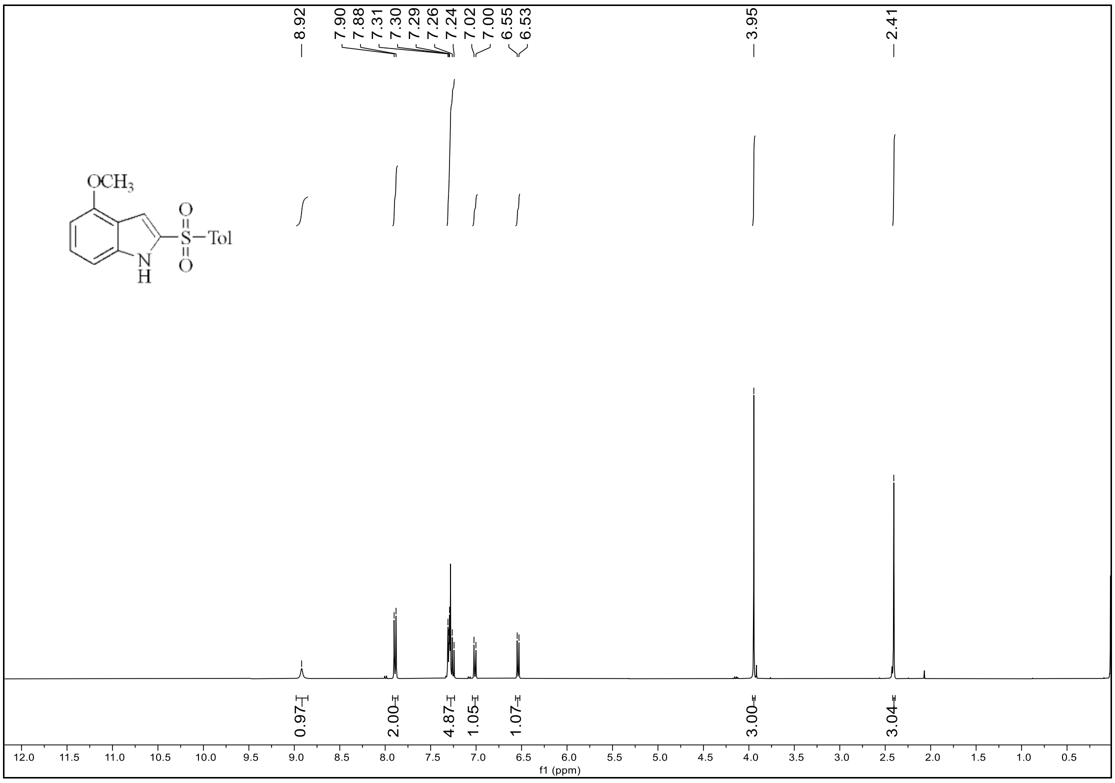

4-(benzyloxy)-2-tosyl-1*H*-indole (3e)

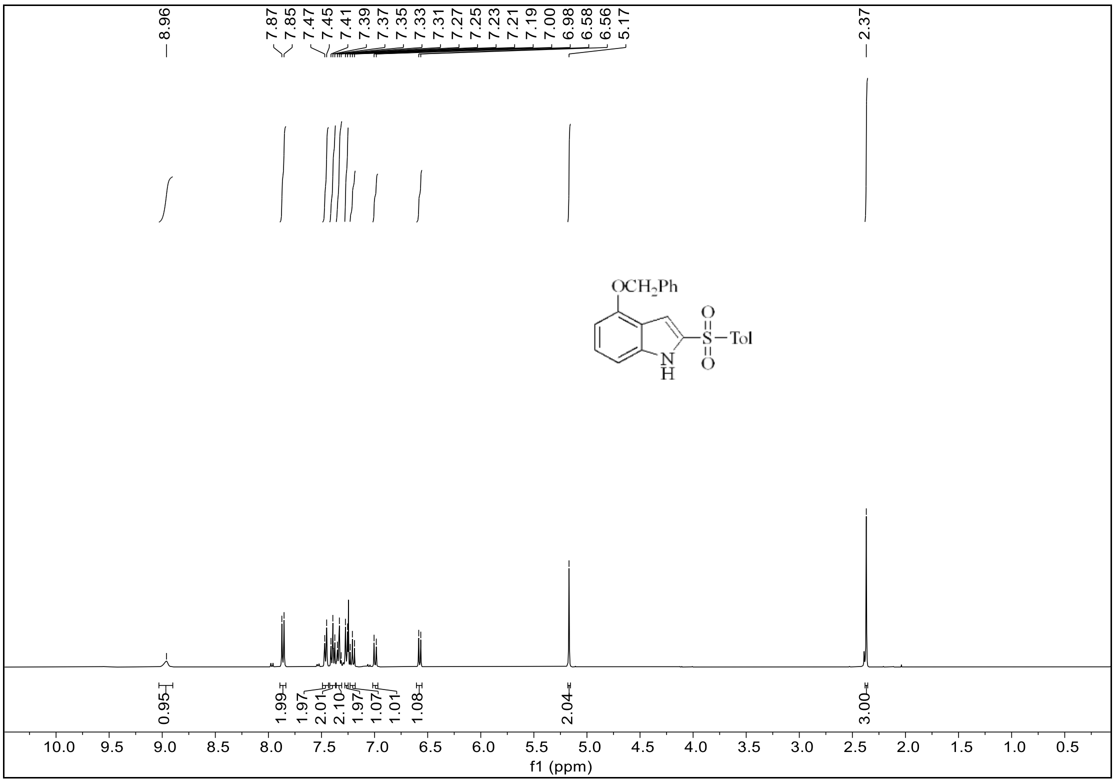

5-methyl-2-tosyl-1*H*-indole (3f)

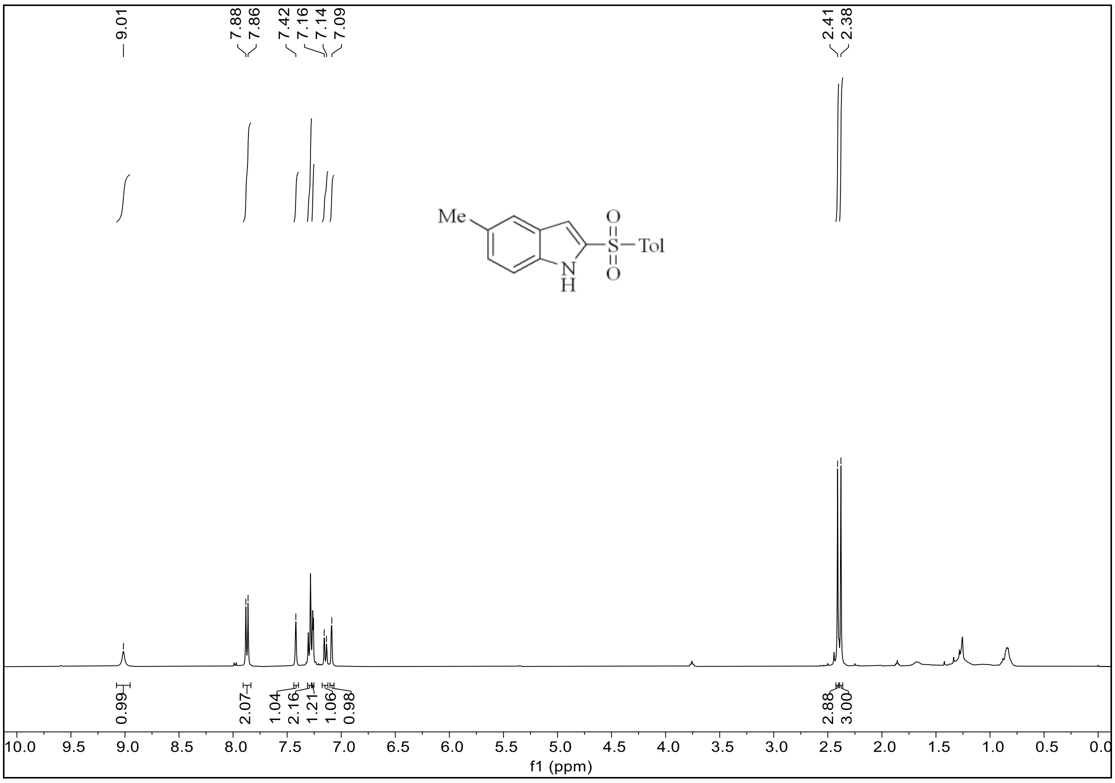

7-methoxy-2-tosyl-1*H*-indole (3i)

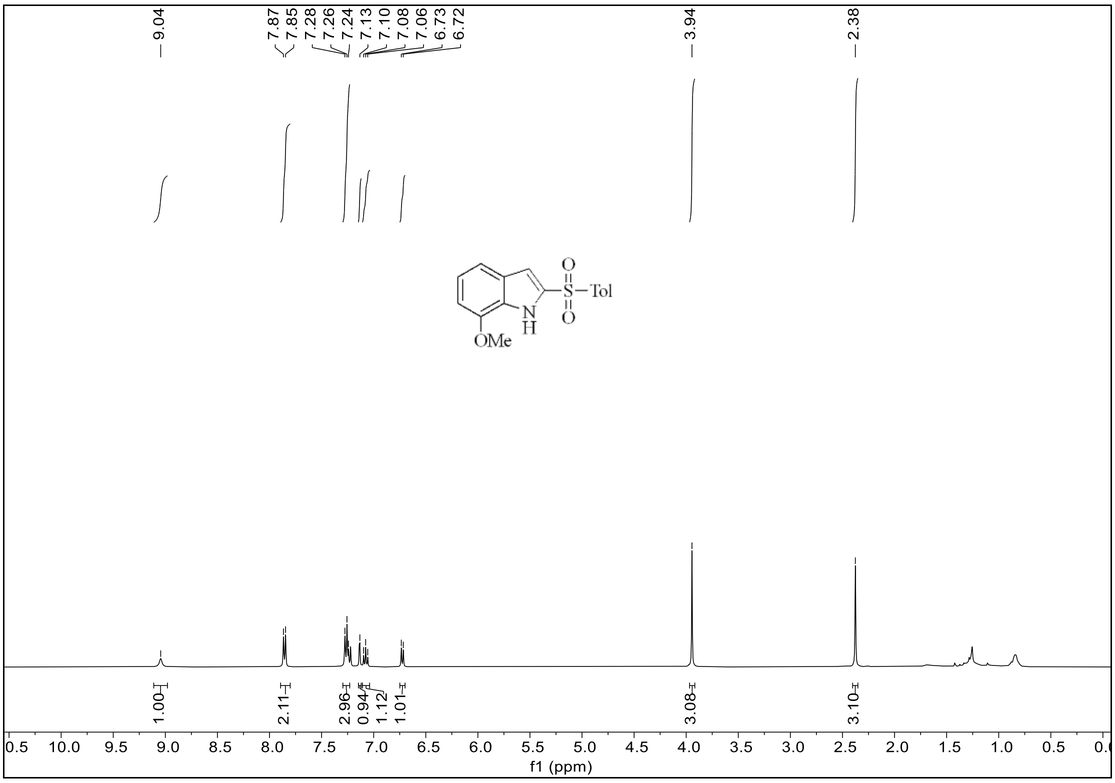

7-bromo-2-tosyl-1*H*-indole (3j)

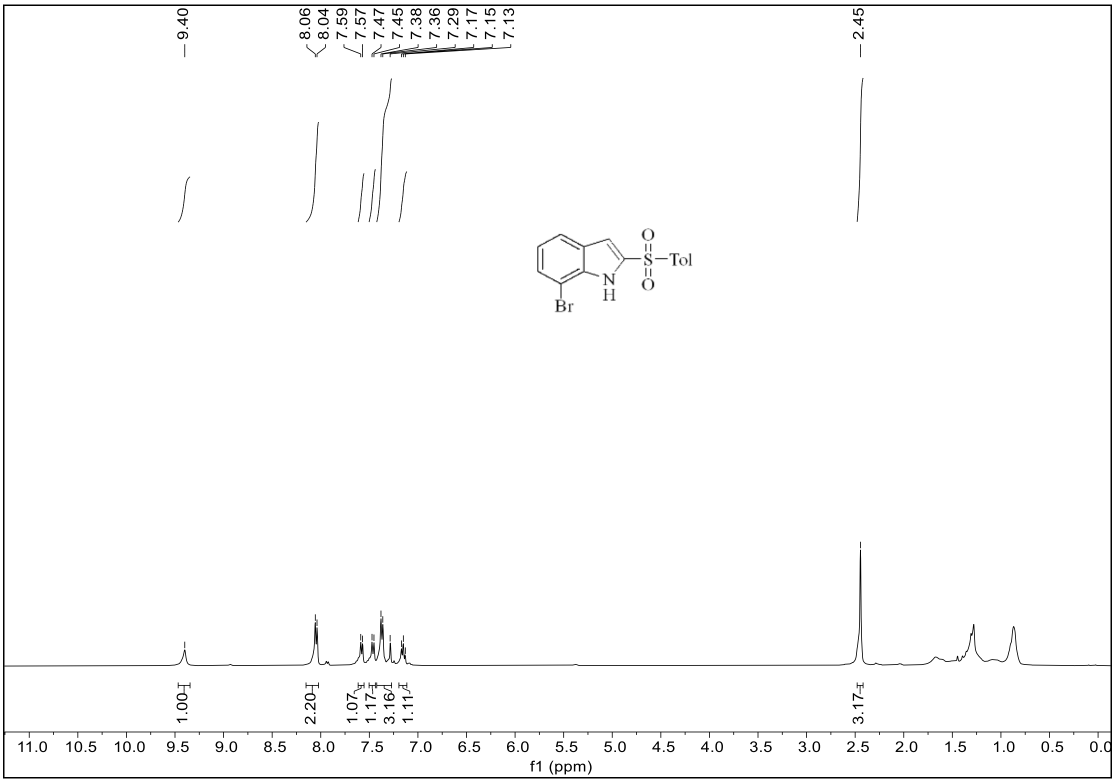

2-methyl-3-tosyl-1*H*-indole (3k)

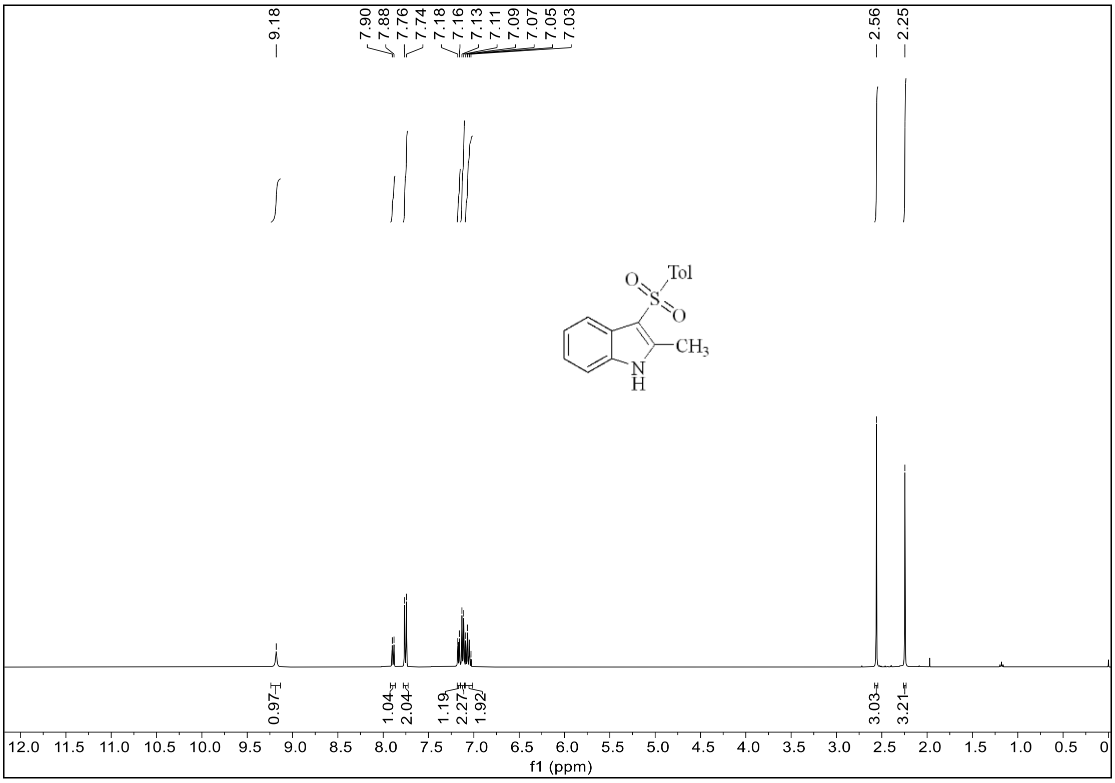

2-((4-(*tert*-butyl)phenyl)sulfonyl)-1-methyl-1*H*-pyrrole (3l)

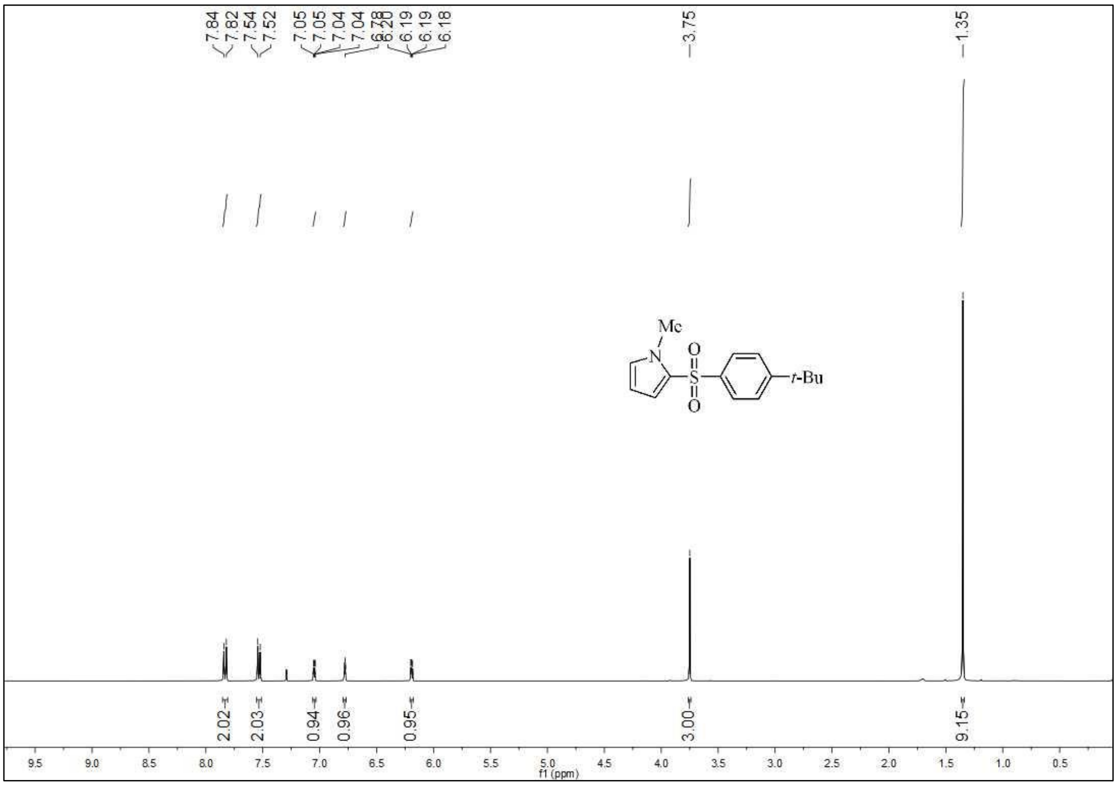

2-(phenylsulfonyl)-1*H*-indole (3n)

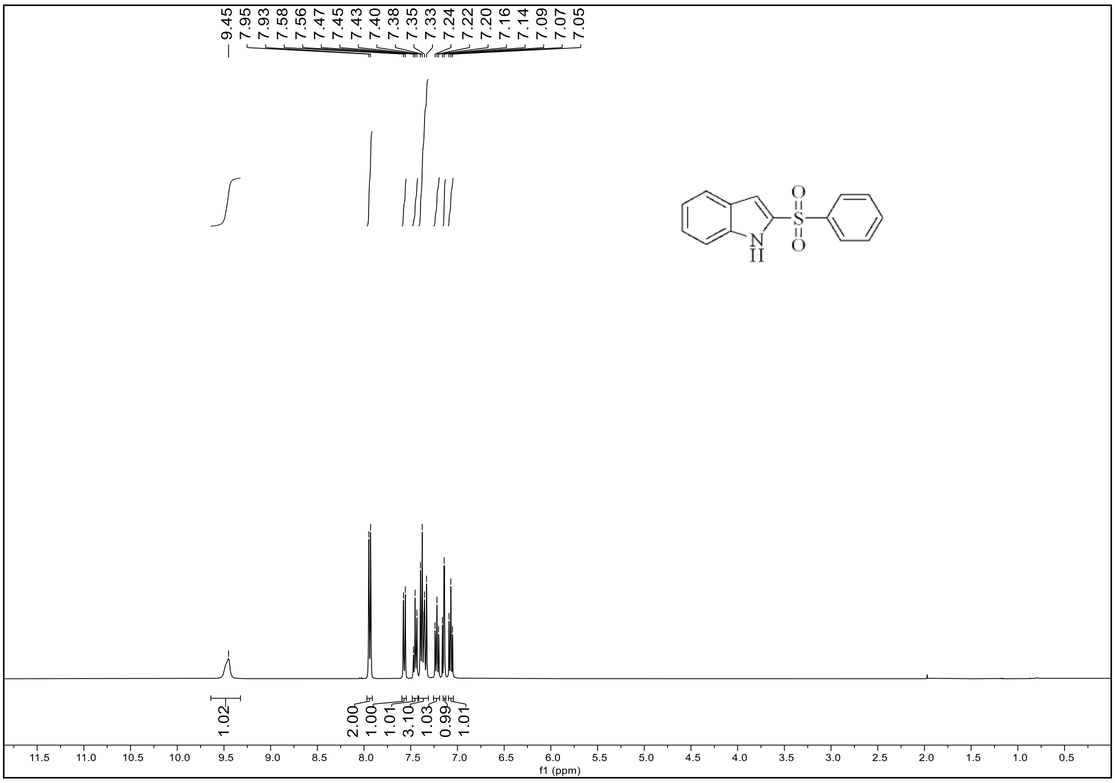

2-((4-methoxyphenyl)sulfonyl)-1*H*-indole (3o)

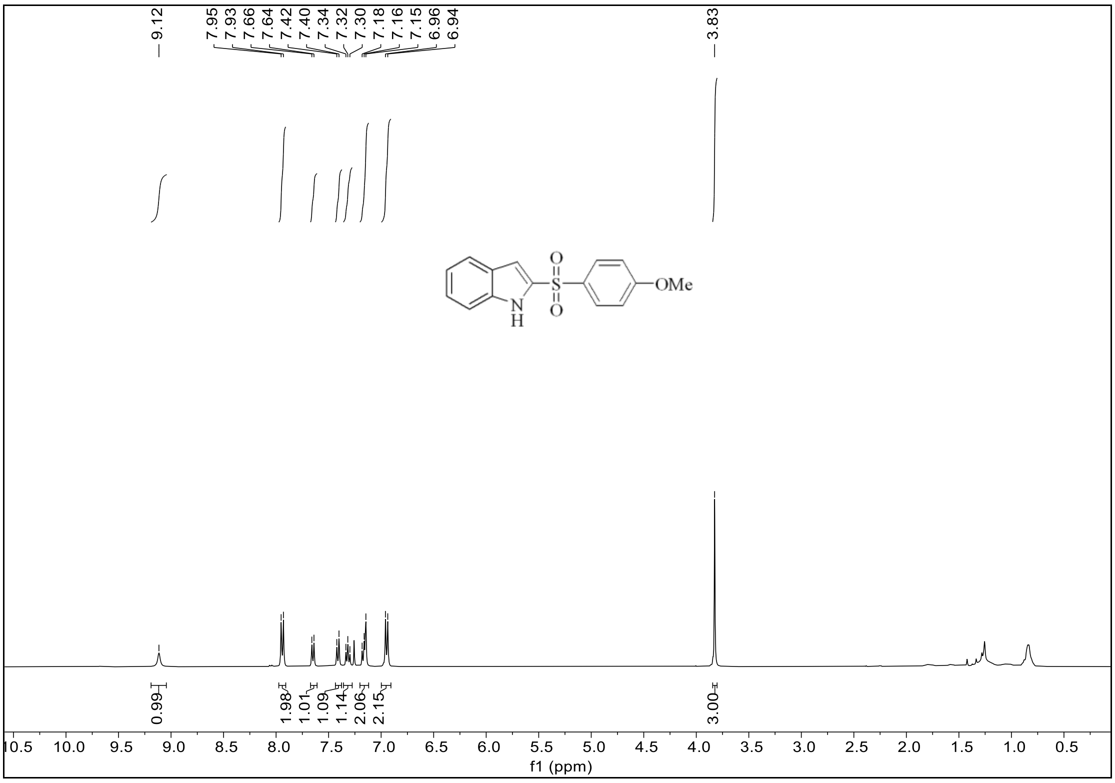

2-((4-(*tert*-butyl)phenyl)sulfonyl)-1*H*-indole (3p)

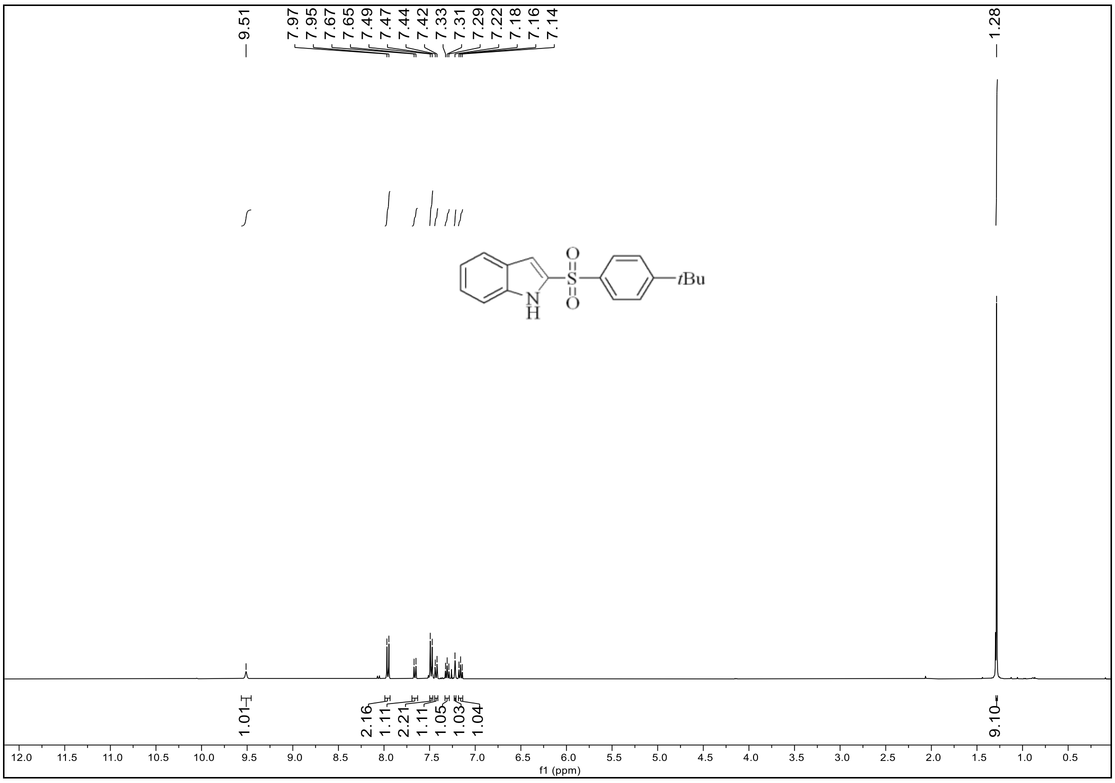

2-((4-fluorophenyl)sulfonyl)-1*H*-indole (3q)

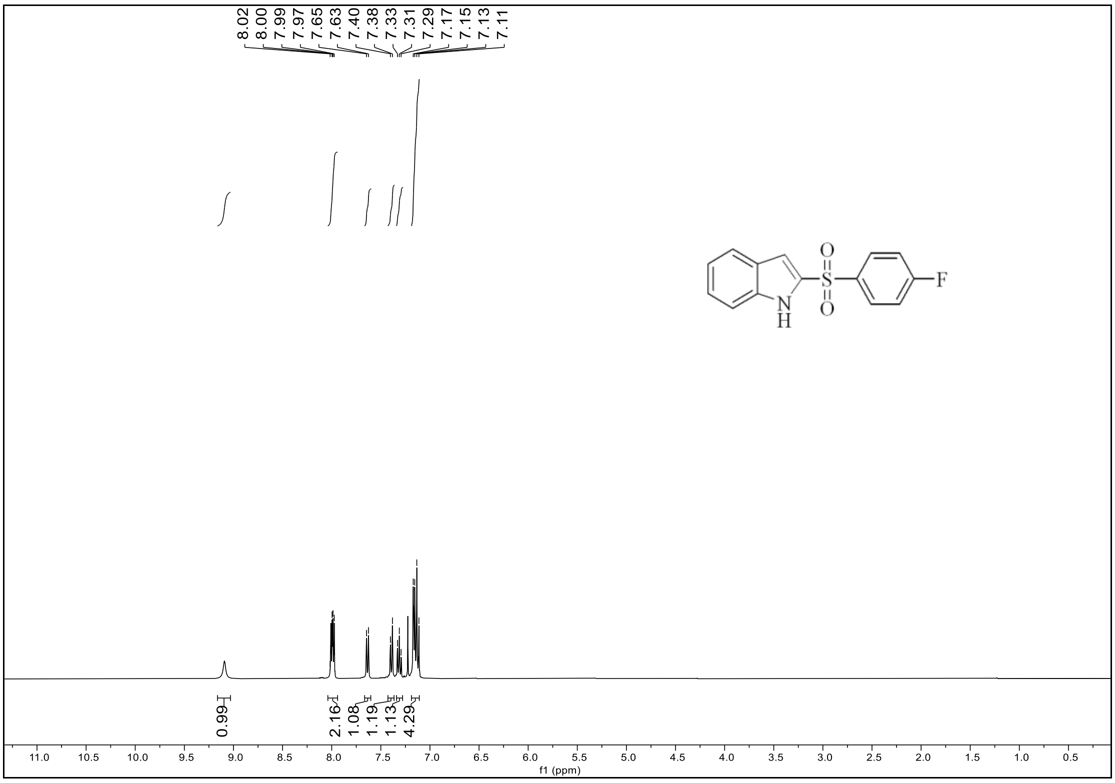

2-((4-chlorophenyl)sulfonyl)-1*H*-indole (3r)

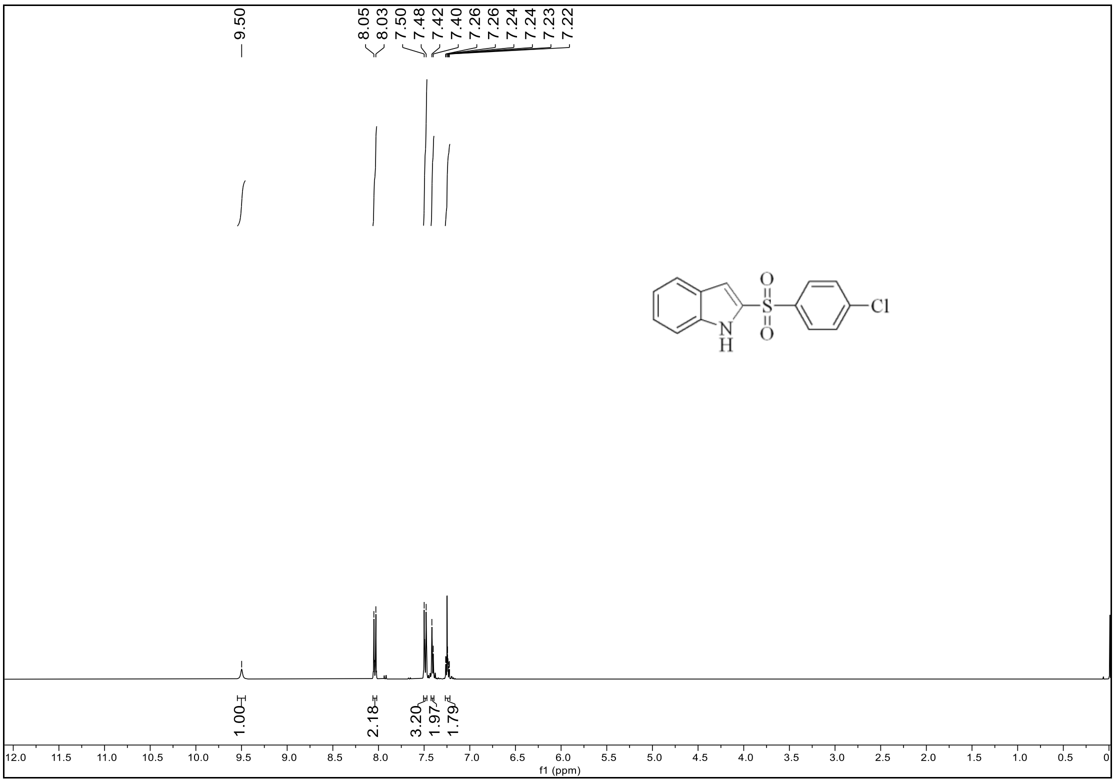

2-((4-bromophenyl)sulfonyl)-1*H*-indole (3s)

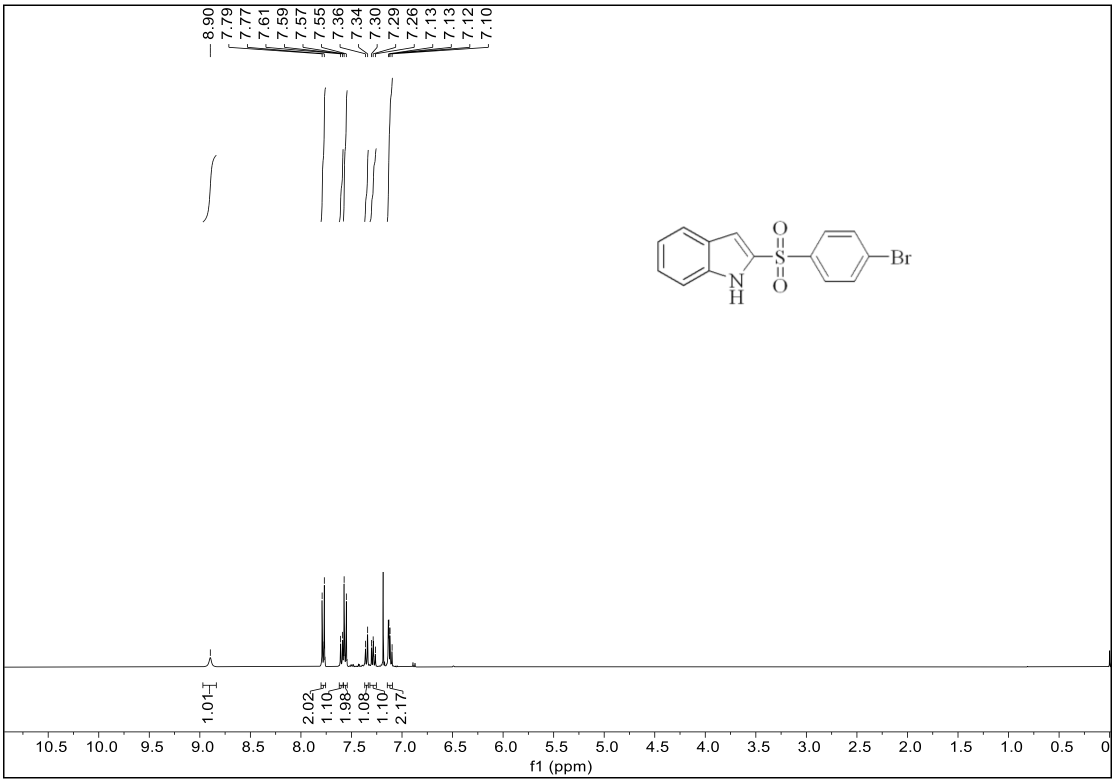

2-((4-(trifluoromethyl)phenyl)sulfonyl)-1*H*-indole (3t)

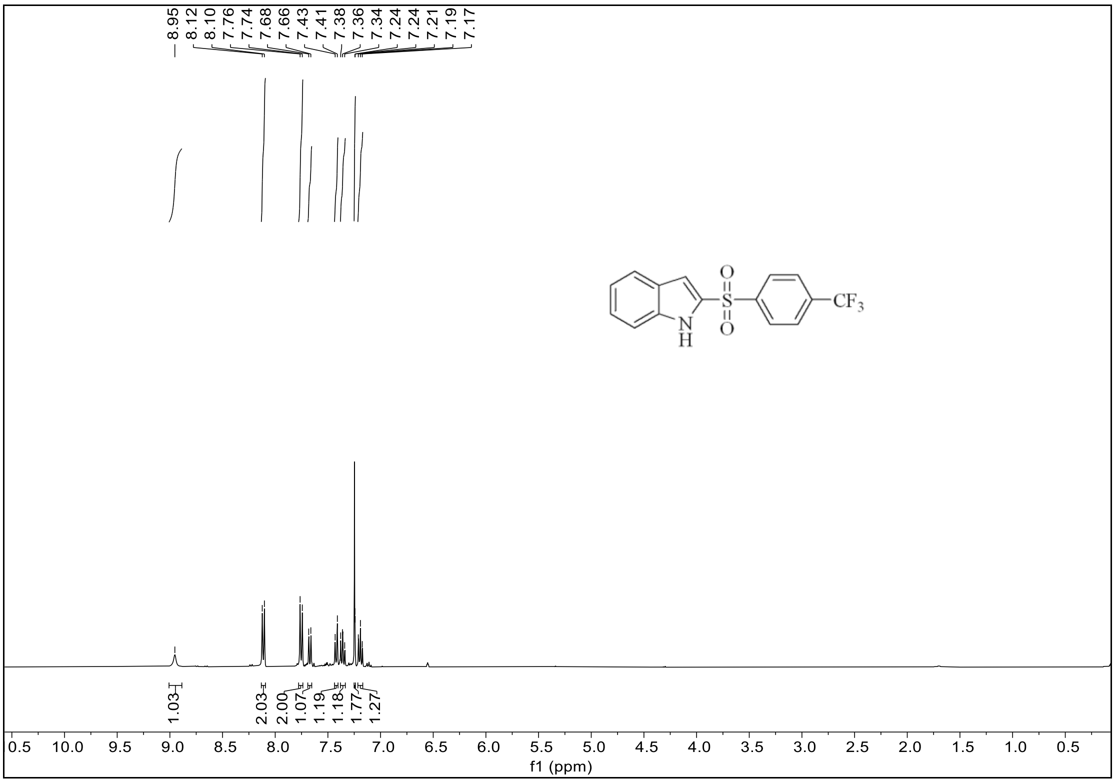

Supplement: Supplementary file 1 [file molecules-29-03564-s001.zip › molecules-3106455-supplementary.pdf]
